# A Polysulfide-Infiltrated Carbon Cloth Cathode for High-Performance Flexible Lithium–Sulfur Batteries

**DOI:** 10.3390/nano8020090

**Published:** 2018-02-07

**Authors:** Ji-Yoon Song, Hyeon-Haeng Lee, Won Gi Hong, Yun Suk Huh, Yun Sung Lee, Hae Jin Kim, Young-Si Jun

**Affiliations:** 1 School of Chemical Engineering, Chonnam National University, 77 Yongbongro, Buk-gu, Gwangju 61186, Korea; jiyoonsong312@gmail.com (J.-Y.S.); rjjdi@naver.com (H.-H.L.); leeys@chonnam.ac.kr (Y.S.L.); 2 Division of Electron Microscopy Research, Korea Basic Science Institute (KBSI), Daejeon 34133, Korea; hongwg79@kbsi.re.kr (W.G.H.); hansol@kbsi.re.kr (H.J.K.); 3 Department of Biological Engineering, Inha University, 100 Inha-ro, Nam-gu, Incheon 22212, Korea

**Keywords:** lithium–sulfur batteries, flexible batteries, carbon cloth, microporous carbon, catholytes

## Abstract

For practical application of lithium–sulfur batteries (LSBs), it is crucial to develop sulfur cathodes with high areal capacity and cycle stability in a simple and inexpensive manner. In this study, a carbon cloth infiltrated with a sulfur-containing electrolyte solution (CC-S) was utilized as an additive-free, flexible, high-sulfur-loading cathode. A freestanding carbon cloth performed double duty as a current collector and a sulfur-supporting/trapping material. The active material in the form of Li_2_S_6_ dissolved in a 1 M LiTFSI-DOL/DME solution was simply infiltrated into the carbon cloth (CC) during cell fabrication, and its optimal loading amount was found to be in a range between 2 and 10 mg/cm^2^ via electrochemical characterization. It was found that the interwoven carbon microfibers retained structural integrity against volume expansion/contraction and that the embedded uniform micropores enabled a high loading and an efficient trapping of sulfur species during cycling. The LSB coin cell employing the CC-S electrode with an areal sulfur loading of 6 mg/cm^2^ exhibited a high areal capacity of 4.3 and 3.2 mAh/cm^2^ at C/10 for 145 cycles and C/3 for 200 cycles, respectively, with minor capacity loss (<0.03%/cycle). More importantly, such high performance could also be realized in flexible pouch cells with dimensions of 2 cm × 6 cm before and after 300 bending cycles. Simple and inexpensive preparation of sulfur cathodes using CC-S electrodes, therefore, has great potential for the manufacture of high-performance flexible LSBs.

## 1. Introduction

Growing demands on electricity storage have inspired tremendous research on rechargeable batteries. As a primary power source, these batteries can supply power to emerging energy storage systems, electric vehicles, and portable electronics, for which the US Joint Center for Energy Storage Research (JCESR) and the US Advanced Battery Consortium (USABC) have set energy density targets of >400 Wh/kg and <$100/kWh [1,2]. Among various battery technologies, lithium–sulfur batteries (LSBs) are at the forefront of meeting these tough requirements. For example, LSBs, consisting of a metallic lithium anode and a chemically active sulfur cathode, have a theoretical energy density of ~2600 Wh/kg and have achieved an energy density of ~500 Wh/kg in prototype cells (Sion Power and Oxis Energy), which is double the gravimetric energy density of state-of-the-art lithium-ion batteries (LIBs, ~240 Wh/kg, the Panasonic NCR18650B) [3]. In addition, sulfur is environmentally benign, earth-abundant, and cheap ($0.02/g), but the practical application of LSBs is hampered by (1) the intrinsic insulating property of charge/discharge products, i.e., sulfur and Li_2_S, and (2) the shuttle effect of soluble intermediates (Li_2_S*_x_*, 2 < *x* ≤ 8) formed by the reaction between lithium and sulfur. These obstacles limit the utilization efficiency of sulfur (lower than 40%) and the charge/discharge cycle stability (less than 100 cycles), respectively, which is why LSBs were initially abandoned after intensive studies in the 1980s [4].

In order to circumvent these technical challenges, innovative strategies have been employed for the last decades in almost all areas of battery development, including electrodes, binders, separators, electrolytes, and cell configuration [5,6,7,8,9,10,11,12]. One of the most prevailing options is arguably to design a sulfur–carbon composite cathode in which the porous conductive carbon does double duty as a sulfur nanoconfinement and current collector [13]. Carbon can easily be tailored to have a micro-structure and texture that are advantageous for electron/ion transfer to the confined sulfur and can be modified with organic/inorganic functionalities like N, O, S, Cu, SiO_2_, and so on, to improve its polysulfide retention ability [14,15,16,17,18]. This strategy significantly improves the utilization of sulfur and the cycle stability of LSBs, but only under certain conditions: (1) The areal sulfur loading is as low as ~2 mg sulfur/cm^2^ electrode, with which cells only yield an areal capacity of 1~2 mAh/cm^2^ at equilibrium. This value is far below that of the commercial LIBs (4 mAh/cm^2^) [19,20]. (2) The sulfur–carbon composite consists of more than 30 wt. % electrochemically inactive carbon support and requires an additional polymer binder, a conductive carbon additive, and a metal current collector for electrode preparation. Such LSBs lose the benefit of lightweight and inexpensive sulfur. (3) The ratio of the electrolyte volume to the sulfur mass is as low as 5~10 μL/mg [9,21,22]. Although utilization of sulfur is limited, an electrolyte-deficient condition suppresses polysulfide dissolution due to the solubility limit and thus prolongs the lifetime of cells. It is, however, not only unclear if the composite cathodes contribute to the high performance, but also questionable that adding a small but proper amount of electrolyte is practical for LSB manufacturing.

For practical application of LSBs, it is thus crucial to develop a sulfur–carbon composite cathode that exhibits high areal capacity and cycle stability for high sulfur loading and excess electrolyte conditions. In particular, composite electrodes that are simple and inexpensive to prepare are essential for LSB manufacturing. Herein, we report that an Li_2_S_6_-infiltrated carbon cloth (CC-S) cathode exhibited a high areal capacity of 4.3 mAh/cm^2^ at C/10 for 140 cycles and 3.2 mAh/cm^2^ at C/3 for 200 cycles with a sulfur loading of 6 mg/cm^2^ and an electrolyte-volume-to-sulfur-mass ratio of ~22 μL/mg. A freestanding CC with a high surface area and uniform micropores performed double duty as a porous support and a current collector. The active material in the form of an Li_2_S_6_ catholyte was simply infiltrated into the CC during cell fabrication. We use a commercially available CC material to avoid multiple steps in the preparation of efficient flexible 3D carbon-based electrodes with a high surface area [23,24,25,26,27]. It was found that the soluble polysulfide species in the micropores of the CC were well retained and were utilized without severe capacity fading in excess electrolyte conditions. Such conditions were transferred to pouch cells, leading to flexible LSB cells that remain intact even after 300 manual bending cycles. 

## 2. Results and Discussions

**Figure 1:**
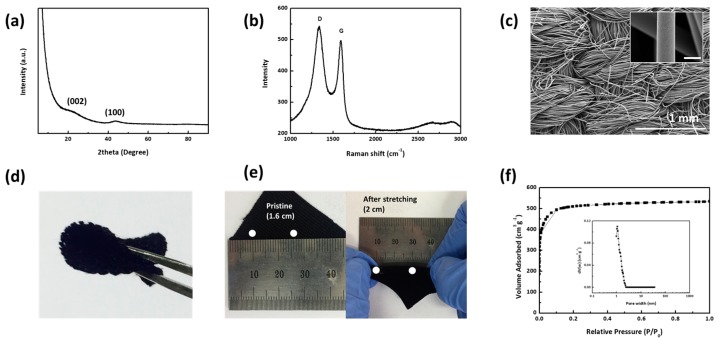
Structural characterization of the carbon cloth (CC). (**a**) XRD pattern; (**b**) Raman spectrum; (**c**) SEM images (scale bar of inset image: 10 µm); photo images of (**d**) folded and (**e**) stretched CC support; (**f**) N_2_ sorption isotherm; and (**f**, inset) the corresponding pore size distribution calculated by the NLDFT method.

CC is a freestanding fabric that solely consists of interwoven bundles of amorphous carbon fibers with a diameter of 10 μm as is observed in an X-ray diffraction (XRD) pattern in Figure 1a, showing two broad peaks at 21.7° and 43.7°, a Raman spectrum with an *I_D_*/*I*_G_ ratio of 1.09 in Figure 1b, and a scanning electron microscopy (SEM) image in Figure 1c. As a current collector, the interlinked structure with high mechanical strength and electrical conductivity provides the confined polysulfide or sulfur with electron and ion conducting channels during the charge/discharge procedure [28,29]. It is also elastic and stretchable (1.25 times) enough to presumably accommodate the volume expansion of sulfur upon lithiation (80%) without losing electrode integrity [21] (Figure 1d,e).

The electrolyte loading (or holding) capacity of CC (63 μL/cm^2^) is much higher than that of the conventional metal current collector (carbon-coated Al, 0.0023 μL/cm^2^), enabling high areal sulfur loading via simple catholyte soaking. As an activated carbon, CC has a high surface area microporous structure, which is efficient in terms of physical ab-/adsorption of both liquid- and solid-phase sulfur species [15,30]. N_2_ sorption analysis, shown in Figure 1f, indicates that CC has a Brunauer–Emmett–Teller (BET) surface area of 1630 m^2^/g and a pore volume of 0.87 cm^3^/g. Based on the pore volume and density of Li_2_S (1.66 g/cm^3^), it is calculated that the maximum sulfur loading capacity in the pores of CC is around 11.5 mg sulfur/cm^2^ CC if we leave 20% of the pore volume empty for electrolyte loading. The theoretical sulfur loading value at 80% pore filling is well above the requirements (≥6 mg sulfur/cm^2^ electrode) to achieve LSB, with an energy density comparable to that of the state-of-the-art LIBs [3,31]. It should be noted that pore size distribution (PSD) calculated by non-local density functional theory (NLDFT) is sharply centered at 1.1 nm (inset image in Figure 1f), which is distinguished from other carbon cloth electrodes previously used for LSBs in that they have a low surface area (~7 m^2^/g) or a hierarchical porous structure where meso- and micro-pores (0.5~2 nm) coexist [28,29,32]. It is also above the range (<0.5 nm) that exerts a strong influence on electrolyte penetration and thereby the electrochemical reaction between polysulfide and lithium [33].

In order to evaluate the electrochemical performance of CC support, cyclic voltammetry (CV), galvano-static cycling with potential limitation (GCPL), and electrochemical impedance spectroscopy (EIS) measurements were conducted and compared with different loadings of Li_2_S_6_ catholytes (2, 6, and 10 mg sulfur/cm^2^ CC). These composite electrodes are designated as CC-S2, CC-S6, and CC-S10, respectively. We used Li_2_S_6_ dissolved in 1 M LiTFSI-DOL/DME (1:1, *v*/*v*) as a sulfur source because it allowed us to skip additional steps in the composite preparation. The total volume of the catholyte remained the same (200 μL), while the Li_2_S_6_ content in the catholyte was changed accordingly, in a range below the calculated maximum at 80% pore filling. The ratio of the catholyte volume to the sulfur mass was about 22 μL/mg, which was higher by a factor of 2~4 compared with the values previously reported. Excess catholyte loading was done to maximize the shuttle effect, allowing us to assess the polysulfide retention ability of the CC support. Note that more than 22 μL/mg results in a significant loss of catholyte during cell crimping and thus in inconsistency in electrochemical performance. 

**Figure 2:**
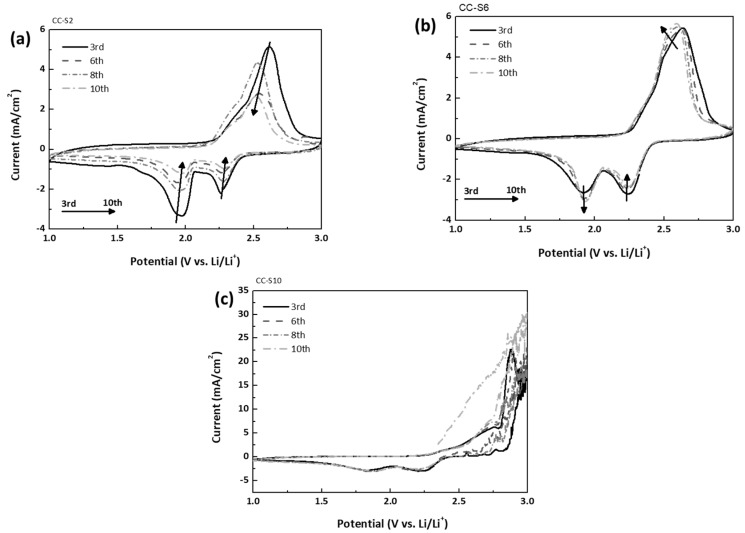
CV diagrams of (**a**) CC-S2; (**b**) CC-S6; and (**c**) CC-S10.

Figure 2 shows CV diagrams of the CC-S electrodes scanned in the potential range of 1.0~3.0 V vs. Li/Li^+^ at a rate of 0.1 mV/s. They feature a typical multi-step redox reaction between lithium and sulfur, showing two sharp cathodic peaks and one broad anodic peak. The peak potential values indeed correlate well with those of typical S_8_ in mesoporous carbon rather than the smaller S_2–4_ in microporous carbon with a pore size around 0.5 nm [13,33]. For example, in CC-S6, the potential scan in the negative direction yields the first cathodic peak around 2.3 V vs. Li/Li^+^ corresponding to the formation of soluble high-order polysulfides (Li_2_S*_x_*, 4 ≤ *x* ≤ 8) from sulfur and the second peak around 1.9 V vs. Li/Li^+^ to their further reduction into the insoluble lithium disulfide (Li_2_S_2_) or lithium sulfide (Li_2_S). The following scan in the positive direction yields a broad anodic peak around 2.6 V vs. Li/Li^+^, indicating the consecutive oxidation from the insoluble sulfides to sulfur.

A gradual decrease in peak current density was observed in CC-S2 (Figure 2a). We attribute this behavior to typical sulfur loss from CC via the well-known dissolution, which can be alleviated by simply increasing the sulfur loading (CC-S6 in Figure 2b and CC-S10 in Figure 2c) [32]. It is notable that the CV diagram of CC-S6 is reproducible over cycles, indicating a stable retention of soluble polysulfides. In addition, CC-S6 had maximum cathodic peak current densities of −2.3 and −3.0 mA/cm^2^ at 2.3 and 1.9 V vs. Li/Li^+^, respectively, translating into a high areal capacity, although polarization between charge and discharge potential was slightly increased due to passivation. A certain amount of Li_2_S deposition is essential because, as a self-trap, it retards the dissolution of sulfur retained in the pores of CC [34]. Further deposition (CC-S10), however, limited the following redox reaction, as was proved by the cathodic peak current densities comparable to those of CC-S6 and the significant over-potential of the anodic peak. It also induced a parasitic reaction with the remaining polysulfides in the catholyte, significantly increasing the anodic peak current density up to 30 mA/cm^2^ [27,35,36,37].

**Figure 3:**
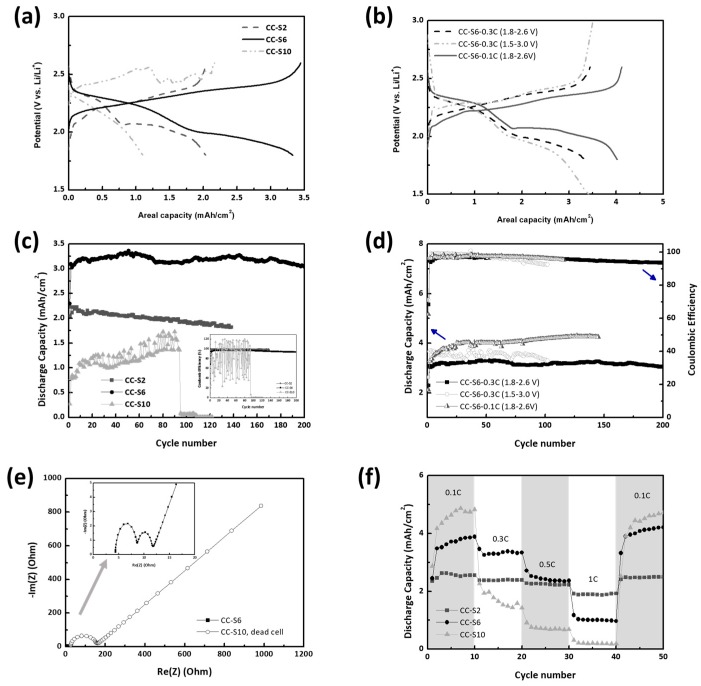
Charge/discharge profiles at the 50th cycle and cycle stability. (**a**,**c**) CC-S2, CC-S6, and CC–S10 operated at C/3 and (**b**,**d**) CC-S6 at C/3 and C/10 in the potential range of 1.8–2.6 and 1.5–3.0 V vs. Li/Li^+^; (**e**) EIS spectra of CC-S6 and CC-S10 after GCPL cycling at C/3; (**f**) Rate capability of CC-S between C/10 and 1 C.

The stable polysulfide retention and high areal capacity of CC were further confirmed by GCPL and EIS analysis (Figure 3). CC-S cells were galvanostatically cycled at C/3 (~500 mA/g sulfur) in the potential range between 1.8 V vs. and 2.6 V vs. Li/Li^+^, before and after which the evolution of impedance was monitored. Given that 500 mA/g exceeds the maximum peak current densities in the CV diagrams and the potential range is slightly narrow to cover the entire redox reaction from solid S_8_ to insoluble Li_2_S, high sulfur loading electrodes such as CC-S6 and CC-S10 are prone to the shuttle effect during GCPL analysis. This again helped us assess the retention ability of soluble polysulfides and thus the cycle stability of CC-S electrodes.

The GCPL results of CC-S electrodes are in good agreement with the CV results. The discharge profile of CC-S6, as expected, shows two distinct plateaus around 2.3 and 1.9 V vs. Li/Li^+^ and the charge profile slopes upward from 2.1 to 2.6 V vs. Li/Li^+^ in which the Coulombic efficiency is close to 100% (Figure 3a,c). It should be noted that the capacity contribution (44%) of the second plateau, corresponding to a reduction from soluble Li_2_S_4_ to insoluble Li_2_S, is much lower than the theoretical value (75%) or that of the CC-S2 (60%). This indicates that the incomplete reduction in CC-S6 mainly resulted from the pore blocking caused by the insoluble sulfides. As mentioned earlier, CC has uniformly developed micropores without transitional meso-/macro-pores that are prone to pore blocking. Once a passivation layer is formed, it not only limits Li^+^ diffusion but also retards the dissolution of soluble polysulfides retained in the pores of CC. Indeed, the capacity contribution remains unchanged in the potential range between 1.5 and 3.0 V vs. Li/Li^+^, a range that is wide enough to cover the entire redox reaction, or slightly increases at C/10 (~160 mA/g), which allows for a more homogeneous deposition of the passivation layer compared to C/3 (Figure 3b,d). Control of Li_2_S deposition is, therefore, the key to a high-performance electrode with high areal capacity and cycle stability. For CC, this can be done by simply changing (or optimizing) concentration of catholyte solution. 

**Figure 4:**
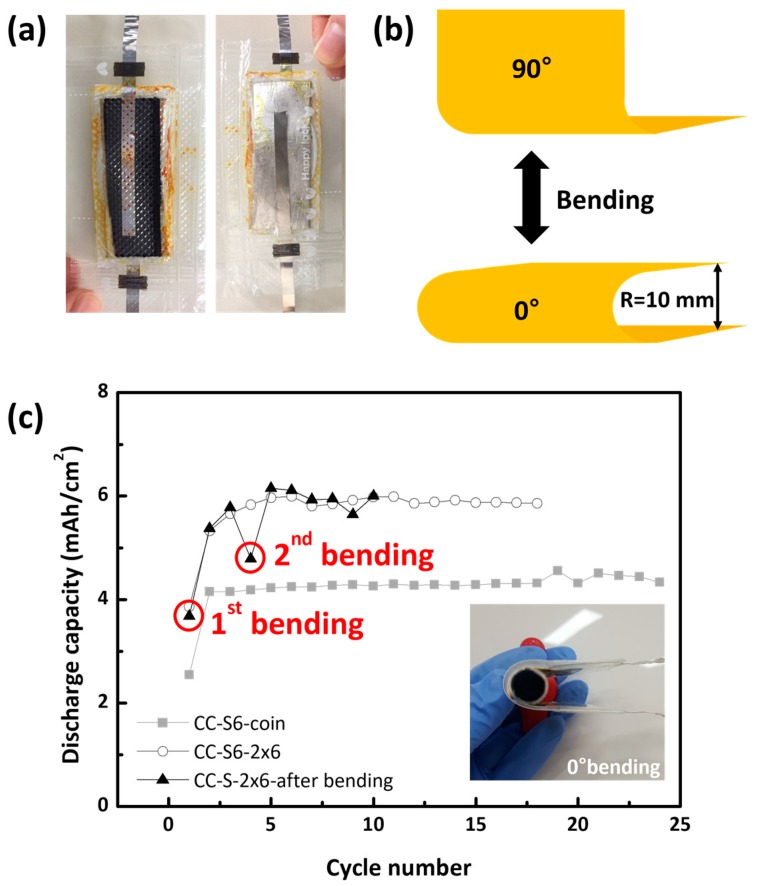
(**a**) Photo images of 2 cm × 6 cm pouch cell showing the CC electrode (left) and the Li metal anode (right); (**b**) Scheme of the pouch cell bending test condition; (**c**) Cycle stability of the pouch and coin cells in the potential range of 1.5–3.0 V vs. Li/Li^+^ at C/10 (inset photo image shows the pouch cell bended at 0°).

CC-S2 showed a gradual decrease in areal capacity from 2.2 mAh/cm^2^, which is typical behavior for low-sulfur-loading cathodes (Figure 3c). CC-S10 suffered from a low areal capacity of 1~1.5 mAh/cm^2^ with poor Coulombic efficiency. It has already been shown in the GCPL profile of CC-S10 that the boundary between two plateaus in the discharge profile becomes blurred due to increased polarization. The higher current applied for CC-S10 precluded further reduction of polysulfides, so the remaining polysulfides induced severe shuttling in the charge procedure. Combined with severe passivation by sulfur or lithium sulfides, this resulted in a Coulombic efficiency fluctuating between 20% and 120%. In contrast, CC-S6 exhibited a stable areal capacity of 3.2 mAh/cm^2^ (547.6 mAh/g) with a capacity loss of 0.03%/cycle, far exceeding those of CC-S2 and CC-S10. This could be further improved to 4.3 mAh/cm^2^ (735.8 mAh/g) without capacity loss at C/10 (Figure 3d). Indeed, Li_2_S deposition was highly reversible in CC-S6 in that the medium frequency semicircle, corresponding to the charge transfer resistance, was well-maintained before and after cycling, while the resistance increase was significant in CC-S10, leading to sudden cell failure (Figure 3e). CC-S6 also had a rate capability that was even better or comparable to that of CC-S2, up to 0.5 C (Figure 3f). In order to glimpse industrial scaling, CC-S6 electrodes were tested in 2 cm × 6 cm pouch cells (Figure 4a). Since the current collection between CC and the Al tab (7 mm width) was inefficient in the presence of the catholyte solution, the pouch cells were cycled at 0.1 C in the potential range of 1.5~3.0 V vs. Li/Li^+^. CC-S6 showed an areal capacity of 6 mAh/cm, which was maintained after 300 bending cycles from 90° to 0° with a bending diameter of 10 mm (Figure 4b,c). To our surprise, the capacity of the pouch cell was higher by about 1.5 times compared with that of the coin cell. We attribute the enhanced performance to the low cell pressure and the efficient Li^+^ dissolution from a Li metal with dimensions of 2 cm × 6 cm.

## 3. Materials and Methods 

### 3.1. Preparation of Electrolytes and Catholytes

The blank electrolyte was 1 M lithium bis(trifluoromethanesulfonyl) imide (LiTFSI, 99.95% trace metal basis from Sigma-Aldrich, Saint Louis, MO, USA) dissolved in 1,3-dioxolane (DOL, 99.8% from Sigma-Aldrich) and 1,2-dimethoxyethane (DME, 99.5% from Sigma-Aldrich) (1:1 volume ratio). Lithium nitrate (LiNO_3_, 99.99% from Aldrich, 1 wt. %) was added to develop a stable solid-electrolyte-interphase (SEI) on the surface of the Li metal anode during cycling. The catholyte (1 M Li_2_S_6_) was prepared by dissolving stoichiometric amount of Li_2_S (99.9% from Alfa-Aesar, Haverhill, MA, USA) and sulfur (≥99.5% from Sigma-Aldrich) in the blank electrolyte.

### 3.2. Cell Assembly

Cells were assembled in an argon-filled glove box with O_2_ and H_2_O level below 0.1 ppm. For CR2032 coin cells, the catholyte was applied on the commercial carbon cloth with a diameter of 14 mm (CH900-20, Kuractive, Kuraray Chemical Co., Ltd., Tokyo, Japan, 21 mg), to which the blank electrolyte was added to make the total amount of catholyte and blank electrolyte 100 µL. Then, the PP separator with a diameter of 19 mm (ceramic coated membrane) was placed on top of the carbon cloth. The blank electrolyte (100 µL) was additionally cast on the separator. Finally, the Li metal anode was placed on top of the separator. 

Procedures for fabrication of the pouch cell were identical to those of the CR2032 coin cell except that pouch cells use a plastic bag for food packaging as a cell case. The catholyte was cast on the CC electrode with dimensions of 2 cm × 6 cm. We used Li metal foil with dimensions identical to those of the CC. Al and Ni tabs as supplementary current collectors were used for CC and Li, respectively. The plastic bag loaded with two electrodes, a catholyte, a blank electrolyte, and a separator was welded using a wireless vacuum packing machine JAS-700 (Point Pack, Ansan, Korea) in the glove box.

### 3.3. Characterization

Powder X-ray diffraction (XRD) pattern was obtained using Rigaku D/max Ultima III (Rigaku, TX, USA) with Cu Kα (Cu-1.8 kW, λ = 0.154 nm) radiation. The morphology of the carbon cloth was observed with field emission scanning electron microscopy (FE-SEM, EX-200, 15.0 kV, Hitachi, Tokyo, Japan) and laser Raman spectrophotometry (NRS-5100, laser wavelength of 531.13 nm, JASCO, Easton, MD, USA). Nitrogen adsorption and desorption isotherms were obtained from Micrometritics ASAP 2020 analyzer (Micromeritics, Norcross, GA, USA) at 77 K. The powder samples were degassed at 150 °C for 12 h under vacuum prior to analysis. Pore size distribution was calculated by non-local density functional theory with an equilibrium model.

The cycle stability and rate capability of CC-S electrodes were evaluated in the potential range between 1.8 and 2.6 V vs. Li/Li^+^ with a WBCS3000 multi-channel cycler (WonATech, Seoul, Korea), unless otherwise noted. Cells were allowed to rest for 6 h prior to analysis in order to completely immerse the electrolyte and catholyte into the pores of CC. Cyclic voltammetry (CV) and electrochemical impedance spectroscopy (EIS) were measured in the potential range between 1 and 3 V vs. Li/Li^+^ and from 300 kHz to 0.05 Hz, respectively, by VSP (Bio-Logic, Seyssinet-Pariset, France). 

The bending test of the pouch cell was performed manually. The pouch cell was bent from 90° to 0° 150 times before cycling. After 3 discharge/charge cycles at C/10, the cell was again bent 150 times and then further cycled.

## 4. Conclusions

**Figure 5:**
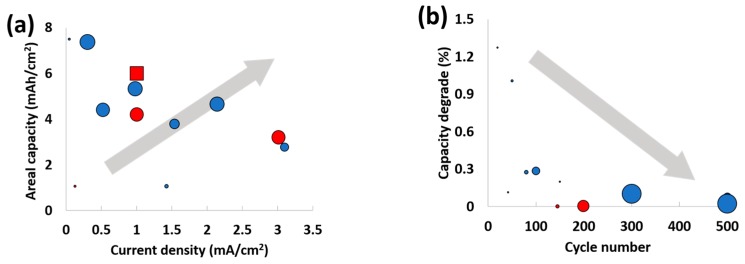
Ragone plots of (**a**) areal current density vs. areal capacity (mean of diameter: amount of sulfur loading) and (**b**) cycle number vs. capacity degrade (mean of diameter: C rate). Circle and square dots mean coin cell and pouch cell data, respectively. Dots in red represent data from this work.

We demonstrated a simple strategy to enable high-performance LSBs in both coin and pouch cell formats. A freestanding, flexible CC with a high surface area and uniform micropores was in situ infiltrated with a catholyte solution and used as a high-sulfur-loading cathode without any additives. LSB cells with the CC-S electrodes showed an improved areal capacity and rate capability at a sulfur loading of 6 mg/cm^2^ and retained its performance after 200 cycles at C/3 in coin cells and after 300 bending cycles in pouch cells. These values are superior to those of the CNT, graphene, and carbon fiber-based freestanding carbon support in terms of areal capacity and cycle stability (Figure 5 and Table 1). We expect that modifying porous structure of CC and/or compositing it with functional materials will further improve its performance, especially at higher sulfur loadings. 

## Figures and Tables

**Table 1:** Electrochemical performance of lithium–sulfur batteries with binder-free 3D electrodes in Figure 5.

No.-	Sample	Current Density	Areal Capacity	Cycle	Capacity Degrade	Sulfur Loading Amount	Reference
		mA/cm^2^	mAh/cm^2^		%	mg/cm^2^	
1	Carbon cloth-Li2S8/GPE	2.144	4.66	500	0.079	6.4	[27]
2	N,S-co-doped graphene sponge	1.541	3.77	100	0.287	4.6	[28]
3	CNT/S	3.01	2.78	500	0.024	3.7	[19]
4	CNT (bottom-up free standing electrode)	0.528	4.41	150	0.198	6.3	[16]
5	CNTs/CC	1.424	1.06	300	0.103	1.7	[23]
6	CFC-S	0.303	7.37	42	0.115	6.7	[29]
7	AFC-S	0.98	5.33	80	0.274	6.5	[21]
8	ACC-Li/S	0.049	7.5	20	1.272	1.27	[30]
9	ACC-S/1M LiNTf2	0.128	1.05	50	1.005	1.27	[31]
10	CC-S6	1.005	4.2	60	0	6	This work
11		3.015	3.2	200	0.03	6	This work
